# Progress of Diseases of the Throat and Nose

**Published:** 1903-08-15

**Authors:** 


### Progress of Diseases of the Throat and Nose.

(*Concluded from page* 333.)

Dr. Bevan[4] believes the best method of operating upon cases of carcinoma of the larynx is with no preliminary tracheotomy. The operation is very briefly as follows: An incision is made from the hyoid to the sternum, the trachea and larynx are dissected carefully out in front and laterally; having put the patient in the Trendelenburg position, the trachea is divided just below the cricoid and stitched into the lower angle of the wound, the mucosa being stitched accurately to the integument by fine horsehair sutures. The entire larynx being removed, the opening into the pharynx and œsophagus is closed by deep-buried sutures. Healing should be by first intention, and the patient is able to swallow and eat. He wears no tracheotomy tube, and Dr. Bevan believes these tubes are a source of great irritation and are probably one of the causes of pneumonia which proves fatal in these cases. In connection with these cases is mentioned "a new method of employing an artificial larynx, as suggested by Gluck, by which a short tracheotomy tube is introduced temporarily into the opening in the trachea, the air carried by a rubber tube, some rubber bands put in vibration along the course of the rubber tube, and a small catheter introduced into the nose, so that the sound is carried into the mouth cavity, enabling the patient to talk or sing without much difficulty." After quoting some statistics collected by Von Bruns, Dr. Bevan summarises as follows: "First, that carcinoma of the larynx early recognised could be removed by complete laryngectomy, or possibly by a less major operation, as a partial laryngectomy or thyrotomy, with a mortality not exceeding 10 per cent. Second, that of the cases which recovered after the operation about half of them would live from one to eight years without recurrence. Third, that if these cases were analysed alongside of those which had not been interfered with, as control cases, one would be led to the conclusion that early operative interference was not only warranted, but dictated by the future history of the cases.

In order to tabulate measurements for operating distances in the nose, Mosher[5] measured 64 half heads, representing at least 50 whole heads; the measurements were made on wet specimens. All measurements were made from the lower edge of the posterior rim of the nasal opening; whilst all vertical measurements were made close to the junction of the septum and the cribriform plate. It is pointed out that if measurements are not made this way, the probe may enter the lateral mass of the ethmoid and the measurements be increased one-eighth to one-quarter of an inch. Mosher gives three measurements for each important point, the middle being the measurement which occurred the greatest number of times, not the mathematical average, the first being the maximum and the last the minimum. The results were as follows:—

| | Inches. | | |
|---|---|---|---|
| To the upper end of the infundibulum... ... | $2\frac{1}{2}$ | 2 | $1\frac{3}{4}$ |
| To the ostium sphenoidale ... ... ... | $2\frac{3}{4}$ | $2\frac{1}{2}$ | $2\frac{1}{4}$ |
| To the posterior wall of the sphenoidal sinus... | $3\frac{3}{4}$ | 3 | $2\frac{1}{2}$ |
| To the lower border of the anterior wall of the sphenoidal sinus ... ... ... ... | $2\frac{5}{8}$ | $2\frac{1}{4}$ | 2 |
| To the ostium maxillare ... ... ... ... | $1\frac{3}{4}$ | $1\frac{1}{2}$ | $1\frac{1}{4}$ |
| The upper end of the infundibulum horizontally to the root of the nose ... ... ... | 1 | $\frac{3}{4}$ / $\frac{3}{8}$ | $\frac{1}{2}$ |

In the last measurement the upper of the two central figures is the average distance, but the working distance is represented by the lower owing to the slope of the posterior wall of the frontal sinus forwards. It is also pointed out that in wet specimens, and in the living, one-eighth to one-quarter of an inch has to be added to the measurements made in the cleaned skull; the latter being made from the lower edge of the nasal notch. Experiments showed that no ratio between the distances in the nose and the various diameters of the skull could be found which would be of any practical importance. It was found, however, that the smallest measurements occurred in heads with toothless jaws. The average distance of the lower opening of the lachrymal duct was about two-thirds of the way to the opening of the antrum, making it one inch. A point of very great importance was noted—viz. that "if a burr is entered at the upper end of the infundibulum and passed directly upward in order to open the frontal sinus, it will in the majority of cases enter the cranial cavity. This is due to the frequent sharp slope forward of the posterior wall of the sinus. In order to enter the sinus from this point, the burr should point forward at an angle of 45°, and would have a working distance of half an inch. In order to bore straight up into the sinus the probe must be held parallel with the teeth and close to them." It was also found in the majority of the specimens that a line passed one quarter of an inch above the lower rim of the orbit, and carried horizontally backwards, passed through the ostium maxillare; a line one quarter of an inch below the rim cut the lower opening of the lachrymal duct; a line one-half an inch below the rim passed through the upper border of the opening of the Eustachian tube. The level of the cribriform plate in 50 cleaned skulls was found to be the mid-point of the inner wall of the orbit; whilst wet specimens showed that this point is one-eighth of an inch above the inner canthus of the eye. It is worthy of note that although the levels of the ostium maxillare, the lower opening of the lachrymal duct, and the Eustachian tube were found to vary, the level of the cribriform plate was "rather constant."

Mayo Collier[6] has made some interesting and original observations respecting the peculiar deformities of the upper jaw, teeth, and palate associated with obstructed nasal respiration. He mentions three of the more common types of this deformity: First, where the lower jaw is prognathous, the arch of the lower jaw being large and surrounding the arch of the upper; the lower incisors are in front of the upper, and the upper molars rest by their outside edges on the lower molars. In the second type, the whole upper jaw is in an extremely atrophic state. The curve of the alveolar arch is less than that of the lower, and the teeth of the upper are inside those of the lower jaw. In addition to this, the palate is found extremely high and the turbinal bones are in close proximity to the septum. In the third variety we have the sides of the upper jaw approximated, but the centre of the arch is thrown forwards, so that the upper incisors overhang those of the lower. The author attributes these deformities to the difference in pressure, owing to impeded nasal respiration, on the outside and inside of the walls of the upper jaw, and in support of this opinion he quotes the results of experiments on young animals, whose nasal cavity or cavities have been obstructed for the purpose of scientific investigation. These results show that "a profound alteration takes place in the development of the upper jaw and a marked alteration in the curves of the alveolar arch and position and height of the palate." The experiments carried out by Professor Ziem, of Dantzic, more than confirm the above results. He finds that obstruction of the nasal cavities tends to a marked arrest of the development of the skull, the whole of the frontal bone as well as the upper jaw being affected. Where only one side was obstructed there was a deviation of the inter-maxillary bone and the sagittal suture to that side, also a lesser length of the nasal bone, of the frontal bone, and of the horizontal plate of the palate bone. The bones were also atrophied, and many other marks of asymmetry were noted. Mayo Collier considers that these remarkable results entirely negative the popular idea that the deformities are hereditary. He does not consider that their production is the result of any muscular action, but that the stream of air passing through the mouth on its way to the larynx and lungs partly abstracts the contents of the nasal chambers and so produces an increased pressure from without on all the walls of nasal box. The corroboration of these views is further manifested by the results obtained by restoring the normal nasal respiration, provided that the deformity is not too marked, and the age of the patient is below that when complete ossification takes place.

**Thyroid.**—Wagner[7] points out the importance of suspecting a primary sarcoma in the thyroid whenever one meets with tumours in bones, and he quotes a case of a woman at 48 with a growth in her femur and an enlarged thyroid. At the autopsy there was in the latter a small nodule having the structure of spindle-celled sarcoma and the growth in the femur was of the same structure. The *Medical Record*[8] in an article on the operative treatment of exophthalmic goitre refers to a series of 74 cases published by the younger Rocher. Of these 59 were operated on, cocaine anæsthesia being used in each case. In some cases one lobe was removed, and in others the thyroid arteries were ligatured. Four of these cases died within ten days of operation with symptoms resembling tetany. Thirty-nine others had well-marked constitutional disturbances such as do not usually follow a considerable surgical operation, viz. tremor, palpitation, sweating, vomiting, etc., and in all these operation was followed by a more or less protracted elevation of temperature and by rapid heart action. The ultimate results, however, were very good, for cure is said to have resulted in 45, and marked improvement in eight others. The writer considers that the treatment should be first operative, and secondly medicinal and hygienic.

[4] Trans. of the Chicago Surg. Soc. [5] Annal Surg., 1902, xxxvi. 554. [6] Lancet, Oct. 18, 1902. [7] Brit. Med. Jour., Epitome. [8] Med. Rec., Nov. 1, 1902.

